# Assessing Als3 Peptide-Binding Cavity and Amyloid-Forming Region Contributions to *Candida albicans* Invasion of Human Oropharyngeal Epithelial Cells

**DOI:** 10.3389/fcimb.2022.890839

**Published:** 2022-07-13

**Authors:** Soon-Hwan Oh, Lois L. Hoyer

**Affiliations:** Department of Pathobiology, College of Veterinary Medicine, University of Illinois at Urbana-Champaign, Urbana, IL, United States

**Keywords:** *Candida albicans*, invasion, fungal adhesion, amyloid-forming region, cytochalasin D, thimerosal, human oropharyngeal epithelial cells, peptide-binding cavity

## Abstract

Although it is widely recognized that disruption of *ALS3* reduces the invasion of *Candida albicans* germ tubes into mammalian oral epithelial cells, the mechanism of this interaction was unexplored. *C. albicans* strains with structurally informed mutations to remove adhesive activity of the peptide-binding cavity (PBC) or aggregative activity mediated by the amyloid-forming region (AFR) were assessed for their ability to invade cultured human oropharyngeal epithelial cells. Initial assays utilized untreated fungal and epithelial cells. Subsequent work used epithelial cells treated with cytochalasin D and *C. albicans* cells treated with thimerosal to investigate invasion mediated by active penetration of germ tubes and epithelial cell induced endocytosis, respectively. Results demonstrated the importance of the PBC for the invasion process: loss of PBC function resulted in the same reduced-invasion phenotype as a *C. albicans* strain that did not produce Als3 on its surface. Invasion *via* active penetration was particularly compromised without PBC function. Loss of AFR function produced a wild-type phenotype in the untreated and thimerosal-treated invasion assays but increased invasion in cytochalasin D-treated epithelial cells. In previous work, reduced AFR-mediated Als3 aggregation increased *C. albicans* adhesion to cultured epithelial cell monolayers, presumably *via* increased PBC accessibility for ligand binding. Collectively, results presented here demonstrate that Als3 PBC-mediated adhesion is integral to its invasive function. These new data add to the mechanistic understanding of the role of Als3 in *C. albicans* invasion into mammalian oral epithelial cells.

## Introduction

*C. albicans* is a common commensal fungus and opportunistic in its ability to cause disease (reviewed in Lopes and Lionakis, 2022). Alterations in host immune status or microbiome can lead to *C. albicans* overgrowth and oropharyngeal candidiasis (OPC) disease manifestations such as surface patches of fungal growth, inflammation, and pain. Swidergall and Filler (2017) reviewed the interplay between human *C. albicans* cells that leads to superficial invasion of the oropharyngeal epithelium. Several *C. albicans* proteins have been referred to as invasins (defined here as proteins that promote *C. albicans* entry into host cells) with Als3 the most-frequently mentioned (Phan et al., 2007; Moreno-Ruiz et al., 2009; Martin et al., 2011; Wächtler et al., 2011; Wächtler et al., 2012; Zhu et al., 2012; Albac et al., 2016; Goyer et al., 2016; Solis et al., 2017; Solis et al., 2018; Phan et al., 2021).

Als3 is one of 8 proteins in the *C. albicans* agglutinin-like sequence family that function in adhesive and aggregative interactions with host cells, abiotic surfaces, and other microbes (reviewed in Hoyer and Cota, 2016). Als3 is present in abundance on the surface of germ tubes and hyphae, providing the opportunity for contact with the *C. albicans* extracellular milieu (Zhao et al., 2004). Adhesive function of Als3 is found in the N-terminal (NT-Als3) domain for which the molecular structure was solved by x-ray crystallography (Salgado et al., 2011; Lin et al., 2014). Adhesive activity (i.e. ligand binding) resides in a peptide-binding cavity (PBC) that can bury up to six C-terminal amino acids of peptides in an extended conformation. Aggregative function is mediated by an amyloid-forming region (AFR) that promotes interactions between Als proteins on the same *C. albicans* cell, on different *C. albicans* cells, and perhaps with similar aggregative features on host or other microbial cells (Lipke et al., 2012). *C. albicans* strains were created to test Als3 PBC and AFR contributions to adhesive phenotype (Lin et al., 2014). Introducing K59M/A116V/Y301F mutations into full-length Als3 and expressing the allele under control of the *ALS3* promoter in a *Δals3/Δals3* background resulted in a strain with the phenotype of an *Δals3/Δals3* null mutant. In other words, even though the K59M/A116V/Y301F strain produced full-length Als3 on its surface, adhesive function was the same as a strain that did not produce any Als3. Introducing I311S/I313S mutations into full-length Als3 abolished the amyloid-forming (aggregative) function of the protein. In fact, decreased Als3 aggregation led to increased adhesion of the I311S/I313S *C. albicans* strain to cultured human epithelial cells, presumably due to increased PBC accessibility to bind ligands (Lin et al., 2014).

*C. albicans* enters (invades) host cells using two general mechanisms called induced endocytosis and active penetration. Wächtler et al. (2012) summarized the differences between these two pathways and detailed a selective inhibition protocol to determine the contribution of each to overall evaluation of invasion. Induced endocytosis, a more passive process, is characterized by rearrangements of mammalian cell microfilaments and hence can be disrupted by treatment with cytochalasin D. Active penetration, or *C. albicans* pushing its way into the mammalian cell and beyond, requires live fungal cells and therefore cannot occur if *C. albicans* is killed by exposure to thimerosal, a treatment that preserves fungal-surface architecture on the dead cells.

Studies of Als3 invasive function have not yet addressed the structural features responsible for this activity. The availability of *C. albicans* K59M/A116V/Y301F and I311S/I313S strains provided the opportunity to test the contribution of the PBC and AFR to *C. albicans* Als3-mediated invasion. An invasion assay method adapted from the work of Park et al. (2005) and Wächtler et al. (2012) was used to assess these structural features in the context of invasion by induced endocytosis and invasion by active penetration of *C. albicans* germ tubes.

## Method

### *C. albicans* Strains

Construction of *C. albicans* strain 1843 (*Δals3/Δals3*) was described by Zhao et al. (2004). Lin et al. (2014) provided detailed methods for construction and validation of the *C. albicans* strains 3464, 3465, and 3467. All *ALS3* alleles were derived from the SC5314 “large allele” (LA) that encodes 12 copies of the 36-amino acid tandem repeat sequence in the central domain of the protein (GenBank accession number AY223552). Oh et al. (2005) previously demonstrated the ability of this allele to mediate *C. albicans* adhesion to monolayers of FaDu human pharyngeal squamous cell carcinoma epithelial cells that were the basis of the invasion assay used here (see below). Strain CAI12 (*ALS3/ALS3*; Porta et al., 1999) was included for comparative immunolabeling purposes only; the strain was a gift from William Fonzi (Georgetown University, Washington, DC, USA).

*C. albicans* isolates were stored at -80°C in 38% glycerol. Strains were streaked to YPD medium (per liter: 10 g yeast extract, 20 g peptone, 20 g glucose, with 20 g Bacto agar for plates) before use and grown to isolated colonies for 24 h at 37°C. A single colony was used to inoculate YPD liquid medium cultures for each experiment. Stock YPD plates were kept at 4°C for no more than 1 week.

### Invasion Assay

The invasion assay was adapted from the methods of Park et al. (2005) and Wächtler et al. (2012). The FaDu human epithelial cell line was purchased from the American Type Culture Collection (catalog number HTB-43) and grown in Minimal Essential Medium (MEM; Gibco) containing 10% fetal bovine serum (Gibco). Growth medium was supplemented with 2 mM L-glutamine and 1 mM sodium pyruvate; no antimicrobials were added. Multiple stock vials were prepared and stored at -80°C. A new vial was thawed for the experiments described here and cells passaged no more than 3 times.

FaDu cells were grown on Permanox Lab-Tek Chamber Slides (Thermo catalog #177437) by adding 3 x 10^5^ cells to each chamber and incubating for 3 days at 37°C and 5% CO_2_ at which time the monolayers were confluent. Fresh culture medium was added to the cells 1 d before the invasion assay.

One colony of each *C. albicans* strain was taken from a YPD stock plate and inoculated into 10 ml of YPD liquid medium in a 50-ml Erlenmeyer flask. Yeast cells were grown to saturation for 16 h at 37°C and 200 rpm shaking. Cells were collected by centrifugation, washed twice in DPBS with Ca^++^ and Mg^++^ (DPBS^+^) and counted using a hemacytometer. Yeast cells were suspended in pre-warmed RPMI 1640 medium with L-glutamine (RPMI; Invitrogen catalog number 11875) at a density of 5 x 10^6^ cells/ml and incubated statically at 37°C and 5% CO_2_ for 1 h. The FaDu monolayers were washed with warm DPBS^+^ and inoculated with 1 x 10^5^ C*. albicans* germ tubes. Inoculated monolayers were incubated at 37°C and 5% CO_2_ for 2 h then washed with 1 ml warm DPBS^+^. FaDu cells and the remaining *C. albicans* germ tubes (presumably sufficiently adherent to withstand the washing step, or already invaded into the FaDu monolayer) were fixed with 4% paraformaldehyde in DPBS^+^ for 10 min, followed by two 1-ml washes with DPBS^+^.

The FaDu/germ tube slide chamber was incubated for 1 h at room temperature with 10 μg/ml Alexa Fluor^®^ 488 conjugate of concanavalin A (ConA; Invitrogen) in DPBS^+^ to label the surfaces of *C. albicans* cells that remained on the monolayer, but outside of FaDu cells. The chamber was rinsed twice with 1 ml each of DPBS^+^, treated with 0.5% Triton X-100 for 10 min to permeabilize the FaDu cells, then rinsed twice with DPBS^+^. The fixed FaDu monolayer was then incubated for 1 h with 10 μg/ml Alexa Fluor^®^ 594 conjugate of ConA in DPBS^+^ (Invitrogen). This label reacted with *C. albicans* cells exposed on the surface of the FaDu monolayer, as well as portions of the cells that were internalized by the FaDu cells during the original 2-h assay incubation period.

The slide chamber was rinsed twice with 1 ml DPBS^+^ and the chambers and gaskets removed from the slide. The slide was covered with ProLong Gold (Invitrogen) and a coverslip added. The slide was imaged on a Nikon A1R confocal microscope to visualize the Alexa Fluor^®^ 488 and Alexa Fluor^®^ 594 dyes. Microscope fields were chosen randomly, starting in the approximate center of the chamber and moving outward to sample different areas of the FaDu monolayer. All cells in each randomly chosen field were evaluated and included in the calculations. Sufficient fields were examined to ensure at least 110 to 150 germ tubes in the analysis. Raw data from the analysis are found in **Supplementary File S1**. Germ tube visualization was aided by Nikon NIS-Elements software. Z-stacked images were displayed at Maximum Intensity Projection and visualized using the ratio view function with the Alexa Fluor^®^ 488 signal as numerator and Alexa Fluor^®^ 594 signal as denominator. The ratio range was from a minimum of 0.05 to maximum of 0.08. Percent invasion (% Inv) was calculated as (# germ tubes invading FaDu cells)/(total germ tubes evaluated) x 100. Number of germ tubes per microscope field was calculated as (total germ tubes evaluated)/(# of microscope fields viewed). **Supplementary File S2** presents a summary of the data.

Invasion was separated into the active penetration and induced endocytosis components by treating FaDu cells with cytochalasin D (Sigma C2618) or treating *C. albicans* cells with thimerosal (Sigma T5125), respectively. For cytochalasin D treatment, a stock solution was prepared in dimethyl sulfoxide (DMSO; Sigma D2438) The FaDu monolayer was rinsed with pre-warmed RPMI and treated with 0.8 μM cytochalasin D in RPMI for 30 min at 37°C and 5% CO_2_. Preliminary experiments ensured that exposure to DMSO did not affect the invasion assay; nearly identical invasion values were obtained for monolayers treated with RPMI and DMSO compared to those only exposed to RPMI (data not shown).

For thimerosal treatment, *C. albicans* DPBS^+^-washed yeast (grown as described above) were resuspended in RPMI at a density of 5 x 10^6^ cells/ml and incubated for 3 h at 37°C and 5% CO_2_. The 3-h time point was used so that germ tubes were grown to the length used for the untreated and cytochalasin D assays (i.e. 1 h pre-growth in a flask, then 2 h growth after addition to the FaDu monolayer = 3 h total). *C. albicans* germ tubes were collected by centrifugation and resuspended in DPBS. Thimerosal was added to 2% final concentration and the germ tubes incubated at room temperature for 1 h with slow agitation. The FaDu invasion assay was completed as described above, using the killed germ tubes. Aliquots of the thimerosal-treated germ tubes were plated on YPD agar and the plates incubated at 37°C for 16 h to ensure that the thimerosal treatment killed the germ tubes.

An additional experimental control involved conducting two experimental replicates where strain 3464 was treated with thimerosal and the FaDu cells treated with cytochalasin D. The combination of treatments should inhibit both active penetration and induced endocytosis. Percent invasion values from the experiments were 0.9% and 0.7% demonstrating the effectiveness of the combined treatments and validating the invasion assay method used (data not shown).

Untreated FaDu invasion assays were conducted in duplicate on four different days. One assay on the second day was discarded due to technical difficulties, leaving a total of 28 data points in the analysis (**Supplementary File S2**). Cytochalasin D experiments were conducted in duplicate on each of three independent days. Thimerosal experiments were conducted in duplicate on each of four independent days. The statistical significance of results was evaluated using a mixed-model analysis of variance (PROC MIXED in SAS; Cary, NC). Separation of means was performed using the LSMEANS option. Data were reported as least squares mean ± standard error of the least squares mean. SAS code and data cards are provided in **Supplementary File S3**.

## Results

*C. albicans* strains that display full-length Als3 under control of the native promoter were described previously (Lin et al., 2014). Strain 3465 [*Δals3/Δals3::ALS3_LA_* (K59M/A116V/Y301F)] encodes Als3 with wild-type surface architecture. However, mutations in its PBC create the structure of Als3 with a bound ligand, even in the absence of a peptide ligand. The Als3 AFR in strain 3465 is not free in solution but associated with the folded portion of the Als3 N-terminal adhesive domain (Lin et al., 2014). Strain 3467 [*Δals3/Δals3::ALS3_LA_* (I311S/I313S)] encodes mutations that abolish Als3 amyloid-forming (aggregative) potential without affecting wild-type surface architecture or conformation of the peptide-binding cavity. Als3 in strain 3464 (*Δals3/Δals3::ALS3_LA_*) is wild-type in sequence and function; the strain is a dosage-matched, positive control for the others in this study. Because both *ALS3* alleles were deleted in strain 1843 (*Δals3/Δals3*; Zhao et al., 2004), it does not produce Als3 on the cell surface.

**Figure 1:**
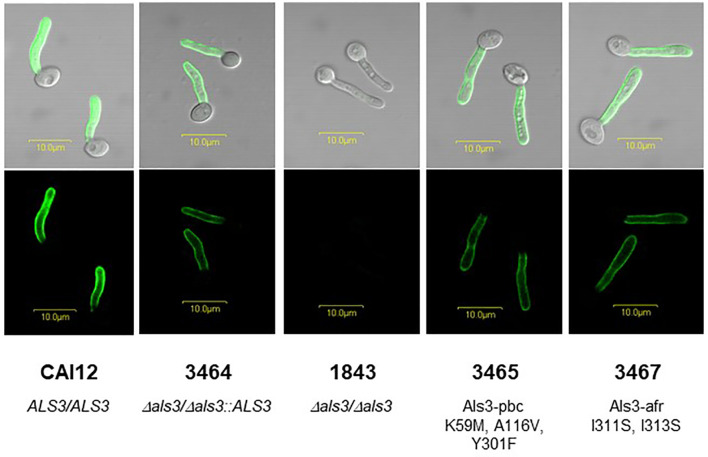
Anti-Als3 immunolabeling of *C. albicans* germ tubes used in this study. The image contains elements of a figure originally presented in Lin et al. (2014) which are reproduced here under the terms of the Creative Commons CC BY license. A single colony each of strains CAI12 (*ALS3/ALS3*), 3464 (*Δals3/Δals3::ALS3_LA_*), 1843 (*Δals3/Δals3*), 3465 (*Δals3/Δals3::ALS3_LA_* (K59M/A116V/Y301F); called Als3-pbc), and 3467 (*Δals3/Δals3::ALS3_LA_* (I311S/I313S); called Als3-afr) were inoculated from YPD stock plates into 10 ml YPD medium in a 50-ml Erlenmeyer flask, and grown for 16 h to saturation at 37°C and 200 rpm shaking. Cells were collected by centrifugation, washed twice in sterile MilliQ water, and counted using a hemacytometer. Cells were released into RPMI medium at a density of 5 x 10^6^ cells/ml and incubated at 37°C for 1 h to form germ tubes. Germ tubes were collected by filtration, fixed in 3% paraformaldehyde, and immunolabeled with anti-Als3 mouse monoclonal antibody 3-A5 and a FITC-conjugated goat-anti-mouse secondary antibody as previously described (Coleman et al., 2009). Fluorescence micrographs showed a similar immunolabeling intensity for all strains that express a single *ALS3* allele under control of the native *ALS3* promoter at the native *ALS3* locus (3464, 3465, and 3467). Strain 1843 did not produce cell-surface Als3 due to complete deletion of both copies of the *ALS3* gene. Strain CAI12 was included to demonstrate increased immunolabeling intensity for a strain that produced Als3 from two intact *ALS3* alleles. Scale bars in each image correspond to 10 μm.

Germ tubes were immunolabeled with anti-Als3 monoclonal antibody 3-A5 that was rigorously validated as recognizing only Als3 (Coleman et al., 2009; **Figure 1**). *C. albicans* CAI12 (*ALS3/ALS3*) was included as a control to display the signal intensity produced by diploid *ALS3* copy number. The similar anti-Als3 labeling intensity for strains 3464, 3465, and 3467 suggested comparable Als3 abundance on the surface of each. Lack of an anti-Als3 signal on the *Δals3/Δals3* null strain 1843 reflected deletion of both *ALS3* alleles (Zhao et al., 2004).

**Figure 2:**
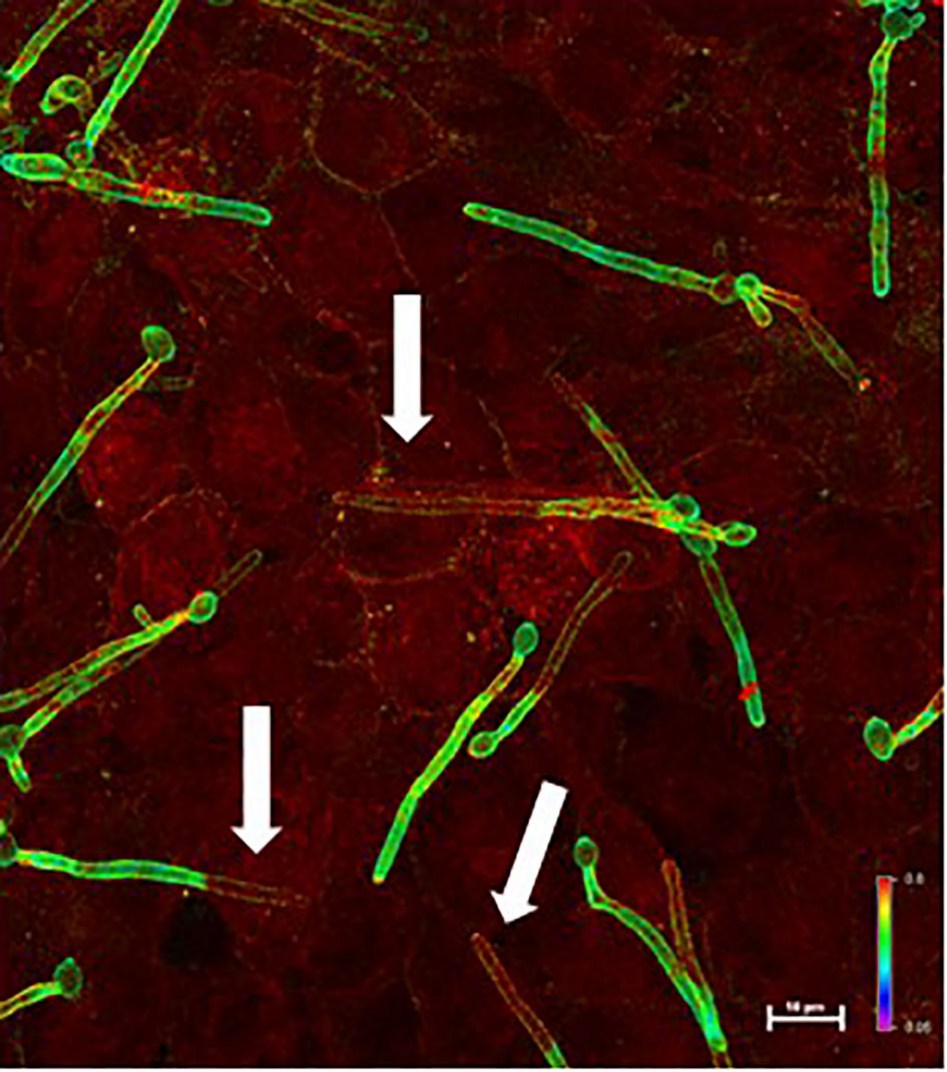
Example micrograph of live *C. albicans* germ tubes invading an untreated FaDu epithelial cell monolayer. Images of epithelial cell monolayers incubated with *C. albicans* germ tubes were captured using a Nikon A1R confocal microscope. Alexa Fluor^®^ 488 and Alexa Fluor^®^ 594 dyes were visualized using the argon ion and helium-neon lasers, respectively. Z-stacked images were analyzed using the ratio view function; a color bar and scale bar are included in the image. Portions of the germ tube that were on the epithelial cell surface (i.e. only labeled with Alexa Fluor^®^ 488-ConA before permeabilizing FaDu cells) appeared green (high 488 signal compared to 594 signal). Red color indicated invading cells that were labeled with both the Alexa Fluor^®^ 488 and Alexa Fluor^®^ 594 dyes. White arrows point to portions of *C. albicans* germ tubes that were below the FaDu monolayer surface.

**Figure 3:**
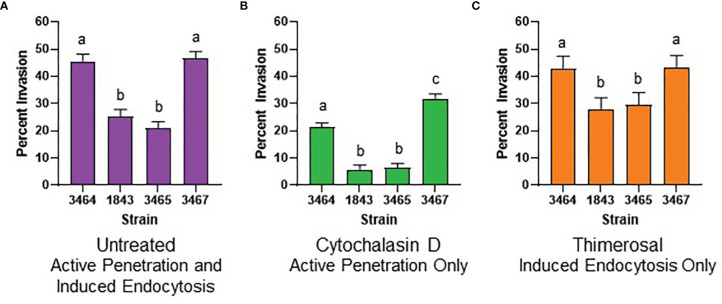
Graphical representation of invasion assay results. **(A)** Histogram displaying results from testing four different untreated *C. albicans* strains on untreated FaDu monolayers. Strains were 3464 (wild-type), 1843 (*Δals3/Δals3*), 3465 (Als3-pbc), and 3467 (Als3-afr). **(B)** Results from testing live *C*. *albicans* germ tubes on a cytochalasin D-treated FaDu monolayer. Invasion was only due to active penetration of the germ tubes into FaDu cells. **(C)** Results from testing thimerosal-killed *C. albicans* germ tubes on an untreated FaDu monolayer to assess invasion by induced endocytosis. For all histograms, least squares means and standard errors of the mean are displayed. Lowercase letters indicate statistical significance groups: for example, strains labeled “a” were not different from each other but were significantly different from strains labeled “b” or “c” (P < 0.05).

Invasion assays used the FaDu human pharyngeal epithelial cell line that was used for the original description of Als3 invasive activity (Park et al., 2005; Phan et al., 2007); the adhesion phenotype with the *C. albicans* mutant strains was already documented (Lin et al., 2014). **Figure 2** shows a representative micrograph of *C. albicans* interacting with the FaDu monolayer. **Figure 3** presents results from testing *C. albicans* strains in the invasion assays.

In the untreated invasion assay (**Figure 3A**), strains 3464 (positive control) and 3467 (Als3-afr) were not significantly different from each other; approximately half of the germ tubes for each strain invaded FaDu cells. *ALS3* deletion (strain 1843) significantly reduced FaDu invasion demonstrating Als3 contribution to the invasive process. Strain 3465 (Als3-pbc) was statistically indistinguishable from 1843. Therefore, display of Als3 that only lacked PBC function reduced invasion to the same degree as a strain that did not display the Als3 protein at all (**Figure 1**).

Previous publications demonstrated that epithelial cell invasion is mediated by induced endocytosis that involves the mammalian cytoskeleton, and active penetration of the epithelial cell by the growing *C. albicans* germ tube tip (Park et al., 2005; Wächtler et al., 2012). Treatment of the FaDu monolayer with cytochalasin D, which disrupted the cytoskeletal network, removed the induced endocytosis component of *C. albicans* invasion, and assessed invasion by germ tube active penetration (**Figure 3B**). For strain 3464, which had one wild-type *ALS3* allele driving production of cell-surface wild-type Als3, approximately 20% of the germ tubes evaluated invaded the FaDu monolayer. Lack of Als3 (strain 1843) or presence of Als3 without a functional PBC (strain 3465) reduced invasion even further and were statistically indistinguishable from each other. Strain 3467, in which the cell-surface Als3 had a functional PBC but reduced aggregative potential due to mutation of its AFR, showed significantly higher invasion compared to the wild-type control 3464.

Treating *C. albicans* germ tubes with thimerosal kills them while maintaining overall cell-surface architecture (Park et al., 2005; Wächtler et al., 2012). Therefore, invasion assessed using thimerosal-treated *C. albicans* cells reflected the induced endocytosis process (**Figure 3C**). In this assay, strains 3464 (positive control) and 3467 (Als3-afr) were statistically indistinguishable. Strains 1843 (*Δals3/Δals3*) and 3465 (Als3-pbc) showed significantly less invasion than the control and were not significantly different from each other.

Germ-tube number per microscope field was used as a proxy for adhesion (**Supplementary Files S1**–**S3**) to more-closely examine the effect of germ tube number on invasion. The overall effect of strain on the number of germ tubes per microscope field was not significant for the untreated (P = 0.06), cytochalasin D-treated (P = 0.16), or the thimerosal-treated (P = 0.69) assay. However, for the untreated assay, the least squares mean value for strain 3467 (16.0 ± 1.1 germ tubes per microscope field) was significantly different in individual comparisons with strain 3464 (12.9 ± 1.1; P = 0.05), strain 1843 (12.8 ± 1.1; P = 0.04), and strain 3465 (11.7 ± 1.1; P = 0.01). In the cytochalasin D-treated assay, strain 3467 (12.9 ± 1.1 germ tubes per microscope field) also showed a significant difference from strain 1843 (10.0 ± 1.1; P = 0.04).

Although strain 3467 tended to have a slightly higher number of germ tubes per microscope field in some of the assays, the data indicated similar numbers of germ tubes for each strain overall in the invasion assays, providing comparable opportunity for each to invade the FaDu monolayer. In all invasion assay configurations, display of Als3 with native structure and surface properties, only lacking PBC function, provided the same invasion phenotype as the *Δals3/Δals3* null strain that did not display surface Als3. These results demonstrated the integral role of the PBC in Als3-mediated invasion of FaDu epithelial cells.

## Discussion

Experiments presented here demonstrate that *ALS3* deletion results in decreased epithelial cell invasion, both by the induced endocytosis and active penetration mechanisms (**Figure 3**). This conclusion concurs with the previous literature that established the importance of Als3 in *C. albicans* epithelial cell invasion (Phan et al., 2007; Moreno-Ruiz et al., 2009; Martin et al., 2011; Wächtler et al., 2011; Wächtler et al., 2012; Zhu et al., 2012; Albac et al., 2016; Goyer et al., 2016; Solis et al., 2017; Solis et al., 2018; Phan et al., 2021). Data presented here also demonstrate that *ALS3* deletion does not eliminate *C. albicans* ability to enter FaDu epithelial cells, suggesting the presence of other *C. albicans* proteins with invasin activity. Ssa1 is often mentioned in this regard and others may exist, as well (Wächtler et al., 2011; Zhu et al., 2012; Phan et al., 2013; Albac et al., 2016; Solis et al., 2017).

The work described here is unique because it tests the contribution of the PBC and AFR to Als3 invasive function (**Figure 1**). Demonstrating indistinguishable phenotypes for the Als3-pbc strain (3465) and the *Δals3/Δals3* null strain (1843) solidifies the role of Als3 PBC-mediated adhesion in the invasion process. This effect is most pronounced in the cytochalasin D assay where *C. albicans* lacking Als3 peptide-binding capability showed only minimal FaDu invasion (**Figure 3B**). Wächtler et al. (2012) suggested that Als3 adhesion provides a solid foothold for the *C. albicans* germ tube to push into epithelial cells *via* active penetration; **Figure 3B** data support that conclusion. Data presented here also demonstrate the role of adhesion in the process of induced endocytosis, indicating biologically meaningful PBC-mediated contact between the FaDu cells and thimerosal-killed *C. albicans* in that assay (**Figure 3C**).

Statistical analysis of the number of germ tubes per microscope field for the various invasion assays indicated little effect of strain. In other words, despite demonstrated differences between the strains in previous adhesion assays (Lin et al., 2014), gentler washing in the invasion assay format likely did not remove all non-adherent germ tubes. An effect of gravity can be imagined for the untreated and thimerosal-treated assays where germ tubes could settle onto the FaDu surface and promote the invasion process without an adhesive contribution from the Als3 PBC. Approximately 25% of thimerosal-killed germ tubes invaded the FaDu monolayer regardless of whether Als3 was absent (1843) or compromised in its PBC (3465; **Figure 3C**). These results emphasize the importance of the PBC in the invasion process, as well as the presence of other molecules that can mediate the interaction in the absence of the Als3 PBC.

Including strain 3467 in the invasion assays tested the role of the AFR and its aggregative function in invasion. Eliminating Als3 AFR-mediated aggregative interactions did not affect FaDu cell invasion in the untreated (**Figure 3A**) or thimerosal-treated assay (**Figure 3C**). However, loss of AFR-mediated aggregation significantly increased the Als3 invasive phenotype in cytochalasin D-treated FaDu cells (**Figure 3B**). Previous evaluation of the adhesive capabilities of strain 3467 showed that loss of aggregative function increased adhesion to FaDu monolayers (Lin et al., 2014). The increased number of strain 3467 germ tubes per microscope field in certain assays is consistent with the possibility of increased adhesion. It was proposed that aggregation of Als3 proteins on the surface of the same *C. albicans* germ tube, or between germ tubes to clump cells, obscures the PBC and reduces Als3 adhesive potential (Lin et al., 2014).

Results presented in **Figure 3** are notable because total invasion percentage (untreated; **Figure 3A**) is not the sum of the percent invasion from the active penetration (**Figure 3B**) and induced endocytosis (**Figure 3C**) assays. These results contrast with ideas presented by Wächtler et al. (2012) that compared directly among the assays and concluded that active penetration is the dominant route of *C. albicans* oral epithelial cell invasion. When viewed from that perspective, results presented here provide the opposite conclusion, prompting additional assessment of differences between the two studies.

One obvious difference between the assays is choice of epithelial cell line: Wächtler et al. (2012) used TR-146 cells instead of FaDu. It is possible that not all epithelial cells produce the same results in the invasion assay (Dalle et al., 2010). Another difference between the assays is the morphology of *C. albicans* cells inoculated onto the epithelial cell monolayers. In the current work, germ tubes of equivalent length were used for all assays. In contrast, Wächtler et al. (2012) inoculated yeast cells into the untreated and cytochalasin D-treated monolayers while germ tubes were used in the thimerosal-treated assay. Yeast cells may be forced by gravity toward the epithelial cell monolayer in a way that perhaps hydrophobic germ tubes may not, resulting in the potential for assay differences depending on the cell morphology used.

Differences in germ tube preparation methods could also affect Als3 abundance, distribution, and interaction with other *C. albicans* cell-surface proteins. In the current work, germ tubes were grown in solution; Wächtler et al. (2012) grew surface-adherent germ tubes then harvested them by scraping from the plastic dish. The authors noted that thimerosal-killed *C. albicans* cells adhered poorly to epithelial cells, an effect not observed in the assays described here. Although adhesion was not specifically quantified here, the assay character did not change between live and killed fungal cells suggesting that low killed-cell adhesion was one reason Wächtler et al. (2012) observed a decreased contribution of induced endocytosis to invasion.

Lastly, two different *als3/als3* strains are described frequently in the literature. Although Wächtler et al. (2012) attributed their strains to Zhao et al. (2004), they are more likely the strains of Nobile et al. (2006). The Zhao et al. (2004) *Δals3/Δals3* strain has a complete deletion of the *ALS3* open-reading frame while Nobile et al. (2006) targeted integration of selectable markers into the 3’ end of *ALS3*, leaving approximately 2.6 kb of the coding region intact. The *ALS3* promoter in this strain should produce a secreted, soluble Als3 protein of approximately 875 amino acids that includes PBC and AFR functions. The effect of this protein on the results of various assays is not known. Wächtler et al. (2012) reported that 3 ± 2% of *Δals3* germ tubes remained attached to the epithelial monolayer, compared to 72 ± 26% for the wild-type control in their untreated invasion assay. This low percentage of *Δals3* cells is considerably different than the appearance of germ tubes in the assays described here and could be caused by production of a soluble adhesion inhibitor.

The idea that Als3 adhesive function is the foundation for its role in invasion is widespread in the literature, but not demonstrated conclusively until now. Data presented here clearly define the role of the Als3 PBC in these activities. These interactions were reviewed by Hoyer and Cota (2016) who hypothesized mechanisms by which Als3 could contact ligands such as cadherins that drive induced endocytosis (Phan et al., 2007) or ligands involved in the cadherin-independent endocytic pathway described by Wächtler et al. (2012). The current work provides the foundation needed to pursue these next questions to better understand interactions between *C. albicans* germ tubes and mammalian oropharyngeal epithelial cells.

## Data Availability Statement

The original contributions presented in the study are included in the article/**Supplementary Material**. Further inquiries can be directed to the corresponding author.

## Author Contributions

LH conceptualized the study, acquired funding, conducted formal analysis, and was responsible for project administration. LH and S-HO developed the study methodology. S-HO performed the investigation. Both authors wrote the original draft of the manuscript and approved the submitted version.

## Funding

This work was supported by discretionary funds available to LH.

## Conflict of Interest

The authors declare that the research was conducted in the absence of any commercial or financial relationships that could be construed as a potential conflict of interest.

## Publisher’s Note

All claims expressed in this article are solely those of the authors and do not necessarily represent those of their affiliated organizations, or those of the publisher, the editors and the reviewers. Any product that may be evaluated in this article, or claim that may be made by its manufacturer, is not guaranteed or endorsed by the publisher.
