# Supplementary material for: Assessing Als3 Peptide-Binding Cavity and Amyloid-Forming Region Contributions to Candida albicans Invasion of Human Oropharyngeal Epithelial Cells
Source: Front Cell Infect Microbiol. 2022 Jul 13;12:890839. doi: 10.3389/fcimb.2022.890839 (PMC9325999; doi:10.3389/fcimb.2022.890839)
Supplement: Supplementary file 2 [file DataSheet_2.docx]

**SUPPLEMENTARY FILE S2 |** Invasion assay data summary*.

| **Untreated** | | | | | | | | | | | | | | | | | |
| --- | --- | --- | --- | --- | --- | --- | --- | --- | --- | --- | --- | --- | --- | --- | --- | --- | --- |
|  |  | **3464** | | | | **1843** | | | | **3465** | | | | **3467** | | | |
| **Day** | **Rep** | **Neg** | **Pos** | **% Inv** | **#F** | **Neg** | **Pos** | **% Inv** | **#F** | **Neg** | **Pos** | **% Inv** | **#F** | **Neg** | **Pos** | **% Inv** | **#F** |
| 1 | 1 | 82 | 54 | 39.7 | 10 | 101 | 46 | 31.3 | 10 | 77 | 28 | 26.7 | 10 | 91 | 61 | 40.1 | 11 |
|  | 2 | 80 | 62 | 43.7 | 10 | 96 | 30 | 23.8 | 10 | 88 | 20 | 18.5 | 10 | 91 | 61 | 40.1 | 10 |
|  |  |  |  |  |  |  |  |  |  |  |  |  |  |  |  |  |  |
| 2 | 1 | 72 | 67 | 48.2 | 10 | 87 | 45 | 34.1 | 10 | 104 | 24 | 18.8 | 10 | 51 | 67 | 56.8 | 5 |
|  |  |  |  |  |  |  |  |  |  |  |  |  |  |  |  |  |  |
| 3 | 1 | 59 | 61 | 50.8 | 10 | 86 | 30 | 25.9 | 10 | 89 | 24 | 21.2 | 10 | 78 | 67 | 46.2 | 10 |
|  | 2 | 57 | 49 | 46.2 | 10 | 89 | 20 | 18.3 | 10 | 77 | 28 | 26.7 | 11 | 78 | 86 | 52.4 | 10 |
|  |  |  |  |  |  |  |  |  |  |  |  |  |  |  |  |  |  |
| 4 | 1 | 89 | 43 | 32.6 | 10 | 110 | 26 | 19.1 | 9 | 108 | 20 | 15.6 | 10 | 97 | 73 | 42.9 | 10 |
|  | 2 | 51 | 67 | 56.8 | 10 | 79 | 24 | 23.3 | 10 | 111 | 23 | 17.2 | 10 | 50 | 43 | 46.2 | 10 |
|  |  |  |  |  |  |  |  |  |  |  |  |  |  |  |  |  |  |
| **Cytochalasin D** | | | | | | | | | | | | | | | | | |
|  |  | **3464** | | | | **1843** | | | | **3465** | | | | **3467** | | | |
| **Day** | **Rep** | **Neg** | **Pos** | **% Inv** | **#F** | **Neg** | **Pos** | **% Inv** | **#F** | **Neg** | **Pos** | **% Inv** | **#F** | **Neg** | **Pos** | **% Inv** | **#F** |
| 1 | 1 | 84 | 38 | 31.1 | 11 | 120 | 7 | 5.8 | 15 | 96 | 7 | 6.8 | 10 | 95 | 43 | 31.2 | 10 |
|  | 2 | 97 | 31 | 24.4 | 10 | 116 | 7 | 5.7 | 10 | 127 | 6 | 4.5 | 10 | 86 | 43 | 33.3 | 10 |
|  |  |  |  |  |  |  |  |  |  |  |  |  |  |  |  |  |  |
| 2 | 1 | 113 | 33 | 22.6 | 10 | 130 | 12 | 8.5 | 10 | 108 | 8 | 6.9 | 10 | 92 | 41 | 30.8 | 10 |
|  | 2 | 97 | 17 | 14.9 | 10 | 114 | 4 | 3.4 | 10 | 119 | 11 | 8.5 | 10 | 103 | 52 | 33.5 | 10 |
|  |  |  |  |  |  |  |  |  |  |  |  |  |  |  |  |  |  |
| 3 | 1 | 96 | 20 | 17.2 | 10 | 85 | 5 | 5.9 | 12 | 126 | 5 | 3.8 | 10 | 61 | 37 | 37.8 | 12 |
|  | 2 | 100 | 22 | 18.0 | 11 | 73 | 4 | 5.2 | 14 | 100 | 9 | 8.3 | 10 | 105 | 34 | 24.5 | 10 |
|  |  |  |  |  |  |  |  |  |  |  |  |  |  |  |  |  |  |
| **Thimerosal** | | | | | | | | | | | | | | | | | |
|  |  | **3464** | | | | **1843** | | | | **3465** | | | | **3467** | | | |
| **Day** | **Rep** | **Neg** | **Pos** | **% Inv** | **#F** | **Neg** | **Pos** | **% Inv** | **#F** | **Neg** | **Pos** | **% Inv** | **#F** | **Neg** | **Pos** | **% Inv** | **#F** |
| 1 | 1 | 92 | 45 | 32.8 | 10 | 79 | 32 | 28.8 | 10 | 93 | 36 | 27.9 | 10 | 75 | 45 | 37.5 | 10 |
|  | 2 | 81 | 64 | 44.1 | 10 | 112 | 25 | 18.2 | 10 | 103 | 60 | 36.8 | 10 | 86 | 38 | 30.6 | 10 |
|  |  |  |  |  |  |  |  |  |  |  |  |  |  |  |  |  |  |
| 2 | 1 | 106 | 40 | 27.4 | 10 | 129 | 35 | 21.3 | 10 | 101 | 29 | 22.3 | 10 | 105 | 50 | 32.3 | 10 |
|  | 2 | 101 | 71 | 41.3 | 10 | 106 | 22 | 17.2 | 11 | 124 | 32 | 20.5 | 11 | 119 | 84 | 41.4 | 10 |
|  |  |  |  |  |  |  |  |  |  |  |  |  |  |  |  |  |  |
| 3 | 1 | 56 | 56 | 50.0 | 10 | 94 | 34 | 26.6 | 10 | 92 | 30 | 24.6 | 10 | 63 | 67 | 51.5 | 10 |
|  | 2 | 63 | 63 | 50.0 | 10 | 91 | 56 | 38.1 | 10 | 99 | 22 | 18.2 | 14 | 69 | 69 | 50.0 | 10 |
|  |  |  |  |  |  |  |  |  |  |  |  |  |  |  |  |  |  |
| 4 | 1 | 103 | 115 | 52.8 | 10 | 114 | 47 | 29.2 | 10 | 99 | 71 | 41.8 | 10 | 67 | 87 | 56.5 | 10 |
|  | 2 | 107 | 88 | 45.1 | 10 | 87 | 64 | 42.4 | 10 | 120 | 96 | 44.4 | 10 | 86 | 76 | 46.9 | 10 |

*The data file was organized and color coded to match **Figure 3**. Rep = experimental replicate. Neg = the number of germ tubes that did not invade the FaDu monolayer among the microscope fields evaluated. Pos = the number of germ tubes that invaded the FaDu monolayer among the microscope fields evaluated. % Inv = percent invasion, calculated as (Pos/(Neg + Pos)) x 100. #F = the number of microscope fields evaluated. Fields were selected randomly as described in **METHOD**. The total number of fields examined was at least 10, and as many as needed to evaluate at least 110 to 150 total germ tubes. Values from this data table were used in the statistical analysis detailed in **Supplementary File S3**.
