# Supplementary material for: Assessing Als3 Peptide-Binding Cavity and Amyloid-Forming Region Contributions to Candida albicans Invasion of Human Oropharyngeal Epithelial Cells
Source: Front Cell Infect Microbiol. 2022 Jul 13;12:890839. doi: 10.3389/fcimb.2022.890839 (PMC9325999; doi:10.3389/fcimb.2022.890839)
Supplement: Supplementary file 3 [file DataSheet_3.docx]

**SUPPLEMENTARY FILE S3 |** SAS code and data cards for statistical analysis.

**A). Assessing strain differences for percent invasion**

options ps=60 ls=100;

data inv;

input day strain$ rep percinv @@;

cards;

(Data cards from below are placed here for each analysis)

;;;

proc print;

run;

proc sort;

by day strain;

run;

proc mixed data=inv;

class day strain;

model percinv = strain;

random day day*strain;

lsmeans strain / pdiff;

run;

**Data cards for untreated assay**

1 3464 1 39.7 1 3464 2 43.7 2 3464 1 48.2 3 3464 1 50.8 3 3464 2 46.2 4 3464 1 32.6 4 3464 2 56.8

1 1843 1 31.3 1 1843 2 23.8 2 1843 1 34.1 3 1843 1 25.9 3 1843 2 18.3 4 1843 1 19.1 4 1843 2 23.3

1 3465 1 26.7 1 3465 2 18.5 2 3465 1 18.8 3 3465 1 21.2 3 3465 2 26.7 4 3465 1 15.6 4 3465 2 17.2

1 3467 1 40.1 1 3467 2 40.1 2 3467 1 56.8 3 3467 1 46.2 3 3467 2 52.4 4 3467 1 42.9 4 3467 2 46.2

**Data cards for cytochalasin D-treated assay**

1 3464 1 31.1 1 3464 2 24.2 2 3464 1 22.6 2 3464 2 14.9 3 3464 1 17.2 3 3464 2 18.0

1 1843 1 5.8 1 1843 2 5.7 2 1843 1 8.5 2 1843 2 3.4 3 1843 1 5.9 3 1843 2 5.2

1 3465 1 6.8 1 3465 2 4.5 2 3465 1 6.9 2 3465 2 8.5 3 3465 1 3.8 3 3465 2 8.3

1 3467 1 31.2 1 3467 2 33.3 2 3467 1 30.8 2 3467 2 33.5 3 3467 1 37.8 3 3467 2 24.5

**Data cards for thimerosal-treated assay**

1 3464 1 32.8 1 3464 2 44.1 2 3464 1 27.4 2 3464 2 41.3 3 3464 1 50.0 3 3464 2 50.0 4 3464 1 52.8 4 3464 2 45.1

1 1843 1 28.8 1 1843 2 18.2 2 1843 1 21.3 2 1843 2 17.2 3 1843 1 26.6 3 1843 2 38.1 4 1843 1 29.2 4 1843 2 42.4

1 3465 1 27.9 1 3465 2 36.8 2 3465 1 22.3 2 3465 2 20.5 3 3465 1 24.6 3 3465 2 18.2 4 3465 1 41.8 4 3465 2 44.4

1 3467 1 37.5 1 3467 2 30.6 2 3467 1 32.3 2 3467 2 41.4 3 3467 1 51.5 3 3467 2 50.0 4 3467 1 56.5 4 3467 2 46.9

**B). Assessing strain differences in the number of cells per microscope field, a proxy for the ability of germ tubes to remain associated with the FaDu monolayer following the invasion assay washing step**

options ps=60 ls=100;

data cells;

input day strain$ rep cellsperf @@;

cards;

(Data cards from below are placed here for each analysis)

;;;

proc print;

run;

proc sort;

by day strain;

run;

proc mixed data=cells;

class day strain;

model cellsperf = strain;

random day day*strain;

lsmeans strain / pdiff;

run;

**Data cards for untreated assay**

1 3464 1 13.6 1 3464 2 14.2 2 3464 1 13.9 3 3464 1 12.0 3 3464 2 10.6 4 3464 1 13.2 4 3464 2 11.8

1 1843 1 14.7 1 1843 2 12.6 2 1843 1 13.2 3 1843 1 11.6 3 1843 2 10.9 4 1843 1 15.1 4 1843 2 10.3

1 3465 1 10.4 1 3465 2 10.8 2 3465 1 12.8 3 3465 1 11.3 3 3465 2 9.5 4 3465 1 12.8 4 3465 2 13.4

1 3467 1 13.8 1 3467 2 15.2 2 3467 1 23.6 3 3467 1 14.4 3 3467 2 16.4 4 3467 1 17.0 4 3467 2 9.3

**Data cards for cytochalasin D-treated assay**

1 3464 1 11.1 1 3464 2 12.7 2 3464 1 14.6 2 3464 2 11.4 3 3464 1 11.6 3 3464 2 11.1

1 1843 1 8.5 1 1843 2 12.3 2 1843 1 14.2 2 1843 2 11.8 3 1843 1 7.5 3 1843 2 5.5

1 3465 1 10.3 1 3465 2 13.3 2 3465 1 11.6 2 3465 2 13.0 3 3465 1 13.1 3 3465 2 10.9

1 3467 1 13.8 1 3467 2 12.9 2 3467 1 13.2 2 3467 2 15.5 3 3467 1 8.2 3 3467 2 13.9

**Data cards for thimerosal-treated assay**

1 3464 1 13.7 1 3464 2 14.5 2 3464 1 14.6 2 3464 2 17.2 3 3464 1 11.2 3 3464 2 12.6 4 3464 1 21.8 4 3464 2 19.5

1 1843 1 11.1 1 1843 2 13.7 2 1843 1 16.4 2 1843 2 11.6 3 1843 1 12.8 3 1843 2 14.7 4 1843 1 16.1 4 1843 2 15.1

1 3465 1 12.9 1 3465 2 16.3 2 3465 1 13.0 2 3465 2 14.2 3 3465 1 12.2 3 3465 2 8.6 4 3465 1 17.0 4 3465 2 21.6

1 3467 1 12.0 1 3467 2 12.4 2 3467 1 15.5 2 3467 2 20.3 3 3467 1 13.0 3 3467 2 13.8 4 3467 1 15.4 4 3467 2 16.2
